# SIRT1 is involved in oncogenic signaling mediated by GPER in breast cancer

**DOI:** 10.1038/cddis.2015.201

**Published:** 2015-07-30

**Authors:** M F Santolla, S Avino, M Pellegrino, E M De Francesco, P De Marco, R Lappano, A Vivacqua, F Cirillo, D C Rigiracciolo, A Scarpelli, S Abonante, M Maggiolini

**Affiliations:** 1 Department of Pharmacy, Health and Nutritional Sciences, University of Calabria, Rende, Italy; 2 Breast Cancer Unit, Regional Hospital, Cosenza, Italy

## Abstract

A number of tumors exhibit an altered expression of sirtuins, including NAD^+^-dependent histone deacetylase silent information regulator 1 (SIRT1) that may act as a tumor suppressor or tumor promoter mainly depending on the tumor types. For instance, in breast cancer cells SIRT1 was shown to exert an essential role toward the oncogenic signaling mediated by the estrogen receptor-*α* (ER*α*). In accordance with these findings, the suppression of SIRT1 led to the inhibition of the transduction pathway triggered by ER*α*. As the regulation of SIRT1 has not been investigated in cancer cells lacking ER, in the present study we ascertained the expression and function of SIRT1 by estrogens in ER-negative breast cancer cells and cancer-associated fibroblasts obtained from breast cancer patients. Our results show that 17*β*-estradiol (E2) and the selective ligand of GPER, namely G-1, induce the expression of SIRT1 through GPER and the subsequent activation of the EGFR/ERK/c-fos/AP-1 transduction pathway. Moreover, we demonstrate that SIRT1 is involved in the pro-survival effects elicited by E2 through GPER, like the prevention of cell cycle arrest and cell death induced by the DNA damaging agent etoposide. Interestingly, the aforementioned actions of estrogens were abolished silencing GPER or SIRT1, as well as using the SIRT1 inhibitor Sirtinol. In addition, we provide evidence regarding the involvement of SIRT1 in tumor growth stimulated by GPER ligands in breast cancer cells and xenograft models. Altogether, our data suggest that SIRT1 may be included in the transduction network activated by estrogens through GPER toward the breast cancer progression.

Estrogens are involved in multiple patho-physiological processes, including the development of diverse types of tumors.^1, 2^ For instance, in breast cancer cells 17*β*-estradiol (E2) triggers stimulatory effects binding to the estrogen receptor-*α* (ER*α*) and ER*β* that regulate the expression of genes which contribute to cell proliferation, migration and survival.^3, 4^ In the last few years, increasing evidence have demonstrated that the G-protein ER (GPER, formerly known as GPR30), can mediate the action of estrogens and certain antiestrogens in both normal and malignant cells.^5, 6, 7, 8, 9^ The ligand binding to GPER induces the release of the membrane-tethered heparin-bound epidermal growth factor, which binds to and activate the epidermal growth factor receptor (EGFR).^10, 11^ Then, the transactivation of EGFR stimulates a transduction network which includes calcium mobilization, MAPK and PI3-K activation in cancer cells and cancer-associated fibroblasts (CAFs), suggesting that GPER may trigger a functional interaction between tumor cells and important components of the tumor microenvironment.^10, 11, 12, 13^ As ascertained by microarray analysis,^10^ GPER regulates a peculiar gene signature involved in the stimulation of estrogen-sensitive malignancies.^7, 10, 14, 15^ In accordance with these findings, GPER has been associated with negative clinical features and poor survival rates in patients with breast, endometrial and ovarian carcinomas.^5^

Recent studies have linked an altered expression of sirtuins family members with several diseases, including different types of tumors.^16^ In particular, the NAD^+^-dependent histone deacetylase silent information regulator 1 (SIRT1) deacetylates several histone and non-histone proteins, leading to the inactivation of tumor-suppressor genes and further target proteins.^16^ SIRT1 influences many hallmarks of longevity, gene silencing, cell cycle progression, differentiation and apoptosis and was found upregulated in a variety of malignancies.^17, 18^ The role of SIRT1 in cancer has been extensively evaluated, however, its potential to act as tumor promoter or suppressor remains controversial.^19, 20, 21^ For instance, SIRT1-mediated deacetylation repressed the functions of several tumor suppressors like p53, p73 and HIC1, suggesting that SIRT1 may be involved in tumor progression.^22, 23^ In contrast, SIRT1 exerted anti-proliferative effects through the inhibition of NF-*κ*B,^24, 25^ a transcription factor having a central role in the regulation of the immune response and carcinogenesis.^26^ As it concerns breast cancer, tumor samples displayed elevated levels of SIRT1 with respect to non-transformed counterparts and the expression of SIRT1 was upregulated by estrogens through ER*α*.^17, 18^ In addition, it was demonstrated that ER*α* physically interacts and functionally cooperates with SIRT1 toward the stimulation of breast tumor cells.^18^ In accordance with these findings, the inhibition of SIRT1 led to the inhibition of ER-mediated signaling, thus indicating that SIRT1 may act as a co-activator of ER*α*.^27^ In the present study, using the GPER-positive and ER-negative SkBr3 breast cancer cells and CAFs obtained from breast cancer patients, we demonstrate that estrogens upregulate SIRT1 expression through the GPER/EGFR/ERK/c-fos/AP-1 transduction pathway. Moreover, we disclose that GPER and SIRT1 have an important role in the pro-survival effects prompted by E2 and the selective GPER ligand G-1 in cancer cells and CAFs treated with etoposide. Noteworthy, SIRT1 contributes to tumor growth elicited by ligand-activated GPER as assessed both *in vitro* as well as in breast tumor xenografts. Collectively, our data provide novel insights into the multifaceted action triggered by estrogenic GPER signaling, which engages also SIRT1, toward breast cancer progression.

## Results

### E2 and G-1 induce SIRT1 expression in ER-negative SkBr3 cells and CAFs

**Figure 1:**
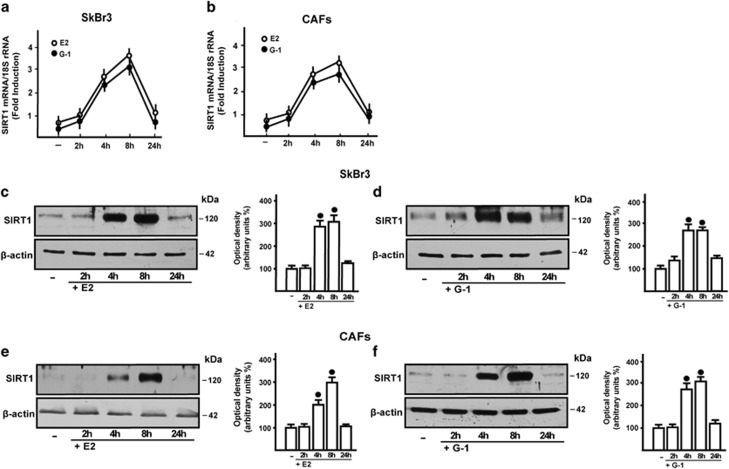
E2 and G-1 induce SIRT1 expression. In SkBr3 cells and CAFs, 100 nM E2 and 1 *μ*M G-1 upregulate the mRNA (**a** and **b**) and protein levels (**c**–**f**) of SIRT1, as evaluated respectively by real-time PCR and immunoblotting. In RNA experiments, gene expression was normalized to 18 S expression and results are shown as fold changes of mRNA expression compared with the cells treated with vehicle (−). Side panels show densitometric analyses of the blots normalized to *β*-actin. Each data point represents the mean±S.D. of three independent experiments. ^•^ indicates *P*<0.05 for cells receiving vehicle (−) *versus* treatments

Previous studies have reported that SIRT1 expression is upregulated by estrogens through ER*α* in breast cancer cells.^10, 18^ Hence, we aimed to evaluate whether estrogens may regulate SIRT1 levels also in ER-negative cancer cells. To this end, we used as a model system the SkBr3 breast cancer cells and CAFs, that are both ER-negative and GPER-positive (Supplementary Figure 1). In time course experiments, E2 and G-1 upregulated SIRT1 expression at both mRNA and protein levels, as determined by real-time PCR (Figures 1a and b) and confirmed by a semi-quantitative PCR evaluation (data not shown).^28^ In line with these results, immunoblotting studies revealed that SIRT1 protein levels are also induced by E2 and G-1 in SkBr3 cells (Figures 1c and d) and CAFs (Figures 1e and f).

### SIRT1 expression is regulated by estrogens through GPER along with the EGFR/ERK/c-fos/AP-1 transduction pathway

**Figure 2:**
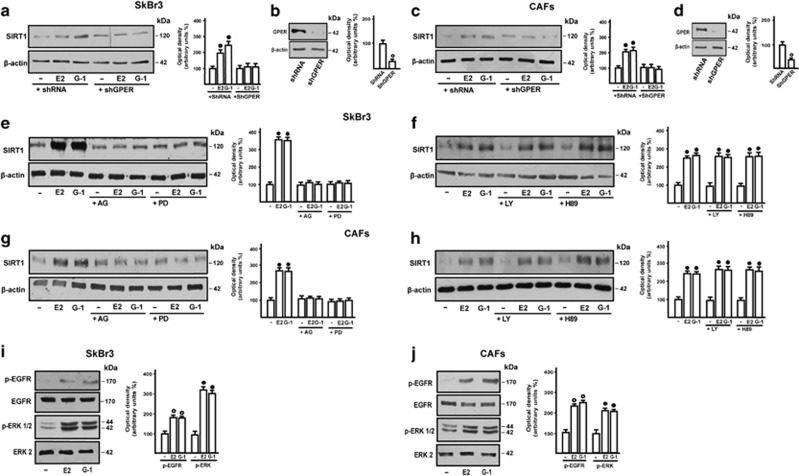
The upregulation of SIRT1 protein levels by E2 and G-1 is mediated by the GPER/EGFR/ERK transduction pathway. SIRT1 protein expression induced by 100 nM E2 and 1 *μ*M G-1 is abolished in SkBr3 cells (**a**) and CAFs (**c**) by silencing GPER with a shGPER construct (**b** and **d**). SIRT1 protein expression in SkBr3 cells (**e** and **f**) and CAFs (**g** and **h**) treated for 8 h with vehicle (−), 100 nM E2 and 1 *μ*M G-1 alone and in combination with 10 *μ*M EGFR inhibitor AG1478 (AG), 10 *μ*M MEK inhibitor PD98089 (PD), 10 *μ*M PKA inhibitor H89, 10 *μ*M PI3-K inhibitor LY294002 (LY), as indicated. ERK1/2 activation and EGFR^Tyr1173^ phosphorylation in SkBr3 cells (**i**) and CAFs (**j**) treated with vehicle (−), 100 nM E2 and 1 *μ*M G-1 for 15 min. Side panels show densitometric analyses of the blots normalized to *β*-actin for SIRT1 expression, ERK2 for p-ERK1/2, EGFR for p-EGFR. Each data point represents the mean±S.D. of three independent experiments. ^•, ○^ indicate *P*<0.05 for cells receiving vehicle (−) *versus* treatments

**Figure 3:**
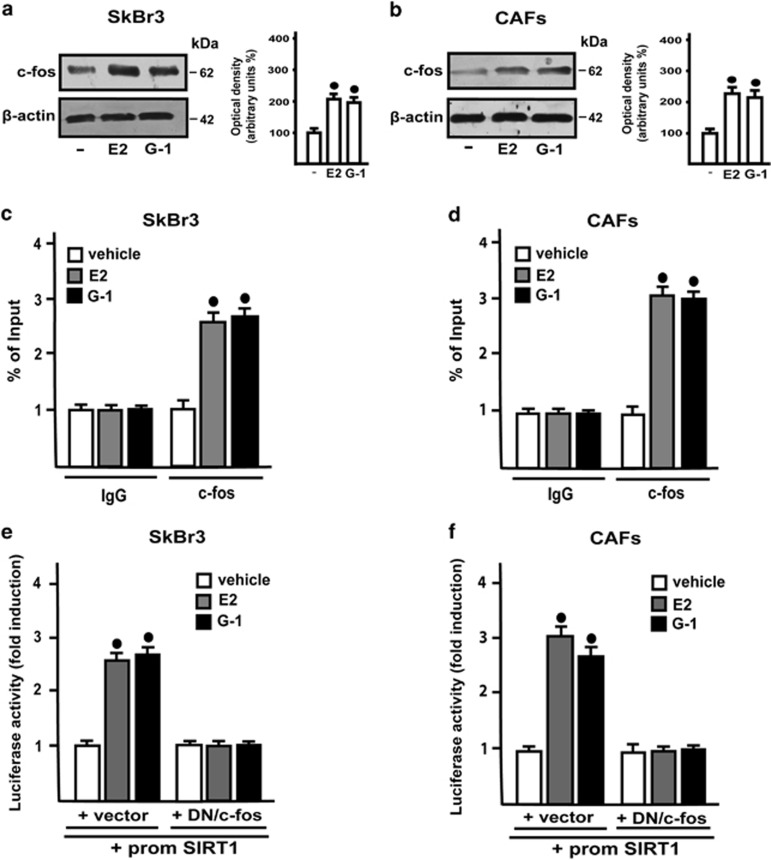
E2 and G-1 induce the expression of c-fos which is recruited to the AP-1 site located within the SIRT1 promoter sequence. In SkBr3 cells (**a**) and CAFs (**b**), the treatment with 100 nM E2 and 1 *μ*M G-1 for 2 h upregulate c-fos, which is recruited to the AP-1 site located within the SIRT1 promoter sequence (**c** and **d**), as ascertained by ChiP assay. The transactivation of the SIRT1 promoter construct induced by an 18 h treatment with 100 nM E2 and 1 *μ*M G-1 is prevented transfecting cells with a construct encoding for a dominant negative form of c-fos (DN/c-fos) (**e** and **f**). In immunoblotting, side panels show densitometric analyses of the blots normalized to *β*-actin. Each data point represents the mean±S.D. of three independent experiments. ^•^ indicates *P*<0.05 for cells receiving vehicle (−) *versus* treatments. Each transfection experiment was performed in triplicate, the luciferase activities from three independent experiments were normalized to the internal transfection control and values for cells receiving vehicle were set as 1 fold induction upon which the activities induced by treatments were calculated

These findings prompted us to evaluate the molecular mechanisms involved in the upregulation of SIRT1 elicited by estrogens in our experimental models. Silencing GPER through a specific short-hairpin GPER construct (shGPER) in SkBr3 cells and CAFs, E2 and G-1 lost the ability to increase SIRT1 expression (Figures 2a and d), suggesting that GPER mediates this effect in both cell types. Next, we found that the upregulation of SIRT1 upon E2 and G-1 treatments is abrogated in the presence of the EGFR inhibitor AG1478 (AG) or the MEK inhibitor PD98059 (PD), whereas the PKA and PI3-K inhibitors, namely H89 and LY294002 (LY), respectively, had no effect (Figures 2e and h). In accordance with these data, E2 and G-1 induced a rapid activation of both EGFR and ERK in SkBr3 cells and CAFs (Figures 2i and j). As the GPER/EGFR/ERK transduction signaling triggers c-fos expression,^6, 13, 15^ we determined the occurrence of this response to E2 and G-1 in both SkBr3 cells and CAFs (Figures 3a and b), then establishing that both ligands prompt the recruitment of c-fos to the AP-1 site located within the promoter sequence of SIRT1 (Figures 3c and d). Further supporting these results, the transactivation of the SIRT1 promoter construct by E2 and G-1 was abolished co-transfecting a dominant negative form of c-fos (DN/c-fos; Figures 3e and f). Taken together, the aforementioned findings suggest that GPER along with the EGFR/ERK/c-fos/AP-1 transduction pathway mediate SIRT1 expression induced by E2 and G-1.

### SIRT1 is involved in the pro-survival effects elicited by estrogens through GPER

**Figure 4:**
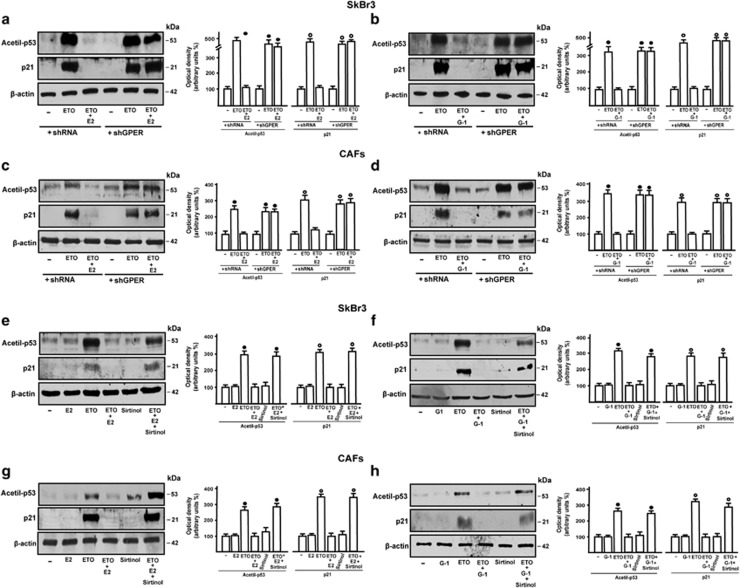
p53 acetylation and p21 upregulation induced by etoposide (ETO) are prevented by E2 and G-1 through GPER and SIRT1. SkBr3 cells (**a** and **b**) and CAFs (**c** and **d**) were transfected with shRNA or shGPER and then treated for 6 h with vehicle (−), 20 *μ*M ETO alone and in combination with 100 nM E2 and 1 *μ*M G-1. Immunoblots showing p53 acetylation at residue Lys382 and p21 protein expression in SkBr3 cells (**e** and **f**) and CAFs (**g** and **h**) treated for 6 h with vehicle (−), 20 *μ*M ETO alone and in combination with 100 nM E2, 1 *μ*M G-1 and 25 *μ*M Sirtinol. Side panels show densitometric analysis of the blots normalized to *β*-actin. Each data point represents the mean±S.D. of three independent experiments. ^•, ○^ indicate *P*<0.05 for cells receiving vehicle (−) *versus* treatments

**Figure 5:**
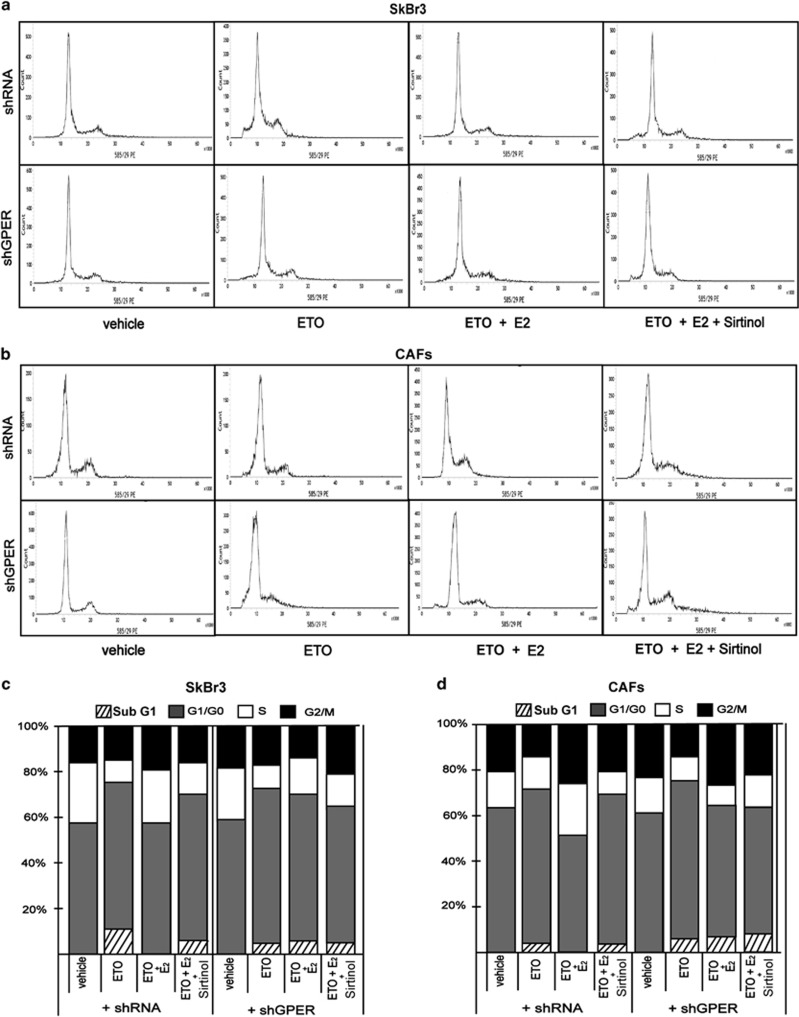
The cell cycle arrest induced by etoposide (ETO) is blunted by E2 via GPER and SIRT1. Cell-cycle analysis performed in SkBr3 cells (**a**) and CAFs (**b**) transfected with shRNA or shGPER for 24 h and then treated for 12 h with 20 *μ*M ETO alone and in combination with 100 nM E2 and 25 *μ*M Sirtinol. (**c** and **d**) histograms show the percentages of cells in subG1, G0/G-1, S and G2/M phases of the cell cycle, as determined by flow cytometry analysis. Values represent the mean±S.D. of three independent experiments

**Figure 6:**
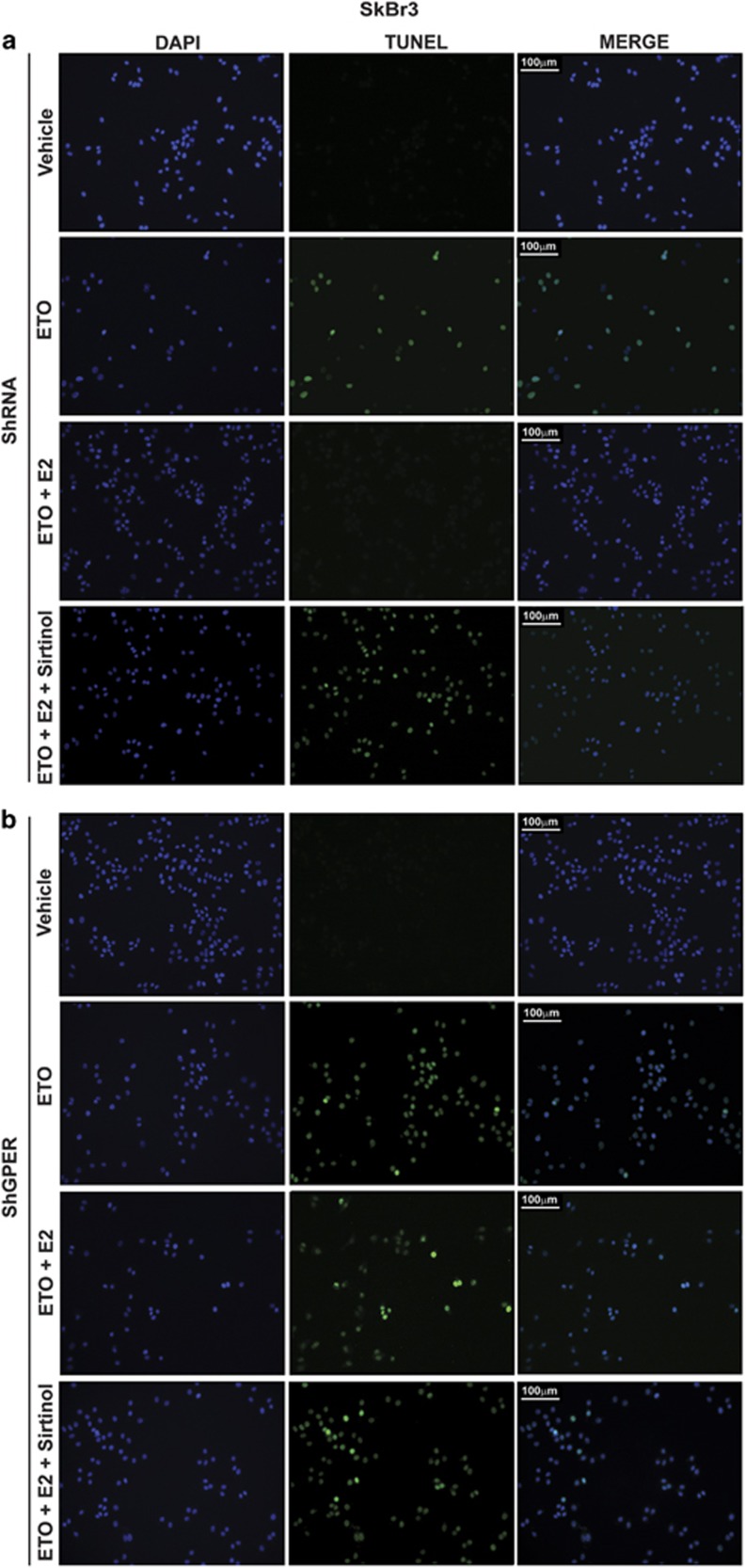
Apoptosis induced by etoposide (ETO) is prevented by E2 via GPER and SIRT1. In SkBr3 cells transfected with shRNA (**a**) or shGPER (**b**), apoptotic changes were detected using Tunel (green) and DAPI (blue) staining after 24 h of treatment with 20 *μ*M ETO alone and in combination with 100 nM E2 and 25 *μ*M Sirtinol. Each experiment shown is representative of 20 random fields. Data are representative of three independent experiments

Previous studies have reported that E2 through ER*α* protects breast cancer cells from oxidative stress and DNA injury.^29^ DNA damage triggers p53 protein acetylation which leads to cell cycle arrest.^30^ This process is mediated by many mechanisms and factors, including the increased expression of the cell cycle inhibitor p21, which facilitates cell accumulation in G0/G-1 phase in order to allow the repair of the damaged DNA.^31^ As p21 expression is controlled by p53 which is regulated by SIRT1, for instance through deacetylation at Lys382 residue,^23^ we investigated the role of SIRT1 in the pro-survival effects elicited by E2 and G-1 via GPER. In this regard, we performed western blot analysis to examine the p53 acetylation at residue Lys382 and the expression levels of p21 in SkBr3 cells and CAFs upon treatment with the DNA damaging agent etoposide (ETO), which was also used in combination with E2 and G-1. As shown in Figures 4a–d, the treatment with E2 and G-1 prevented the activation of p53 and the increase of p21 protein levels triggered by ETO. Of note, this effect was abrogated in both cell types silencing GPER expression by a shGPER construct (Figures 4a and d and Supplementary Figure 2) or treating cells with the SIRT1 inhibitor namely Sirtinol (Figures 4e and h). Next, we performed cell cycle analysis determining that E2 prevents cell cycle arrest induced by ETO in SkBr3 cells and CAFs, however, this effect was no longer evident silencing GPER or in the presence of Sirtinol (Figures 5a and d). Then, we analyzed by TUNEL assay the involvement of GPER and SIRT1 in the pro-survival effects elicited by E2 in ETO-induced apoptosis. The DNA fragmentation induced by ETO was prevented treating with E2 both SkBr3 cells (Figure 6) and CAFs (Supplementary Figure 3), however the effect of E2 was abrogated silencing GPER, using the SIRT1 inhibitor Sirtinol or silencing SIRT1 expression with shSIRT1 (Supplementary Figure 4). Collectively, these findings suggest that GPER and SIRT1 contribute to the protective effects of estrogens upon exposure to the DNA damaging agent ETO.

### GPER and SIRT1 promote tumor growth both *in vitro* and *in vivo*

**Figure 7:**
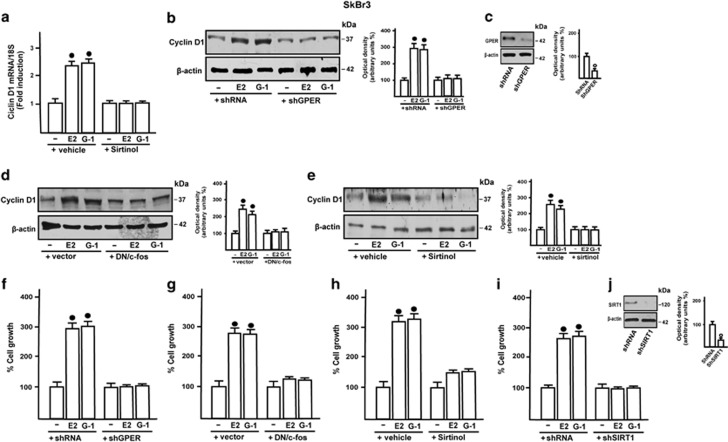
SIRT1 mediates the proliferative effects induced by E2 and G-1 in SkBr3 cells. (**a**) Evaluation of Cyclin D1 mRNA expression upon exposure to 100 nM E2 and 1 *μ*M G-1 alone and in combination with 25 *μ*M Sirtinol. The upregulation of Cyclin D1 protein levels by 100 nM E2 and 1 *μ*M G-1 was abolished transfecting cells with shGPER (**b** and **c**), with the DN/c-fos construct (**d**) or treating cells also with 25 *μ*M Sirtinol (**e**). Cell proliferation induced by 100 nM E2 and 100 nM G-1 was abrogated transfecting cells with shGPER (**f**), with the DN/c-fos construct (**g**), treating cells with 25 *μ*M Sirtinol (**h**) or transfecting cells with shSIRT1 (**i**). In RNA experiments, gene expression was normalized to 18 S expression and results are shown as fold changes of mRNA expression induced by treatments with respect to cells treated with vehicle (−). In immunoblots experiments side panels show densitometric analyses of the blots normalized to *β*-actin. Each data point represents the mean±S.D. of three independent experiments. ^•, ○^ indicate *P*<0.05 for cells receiving vehicle (−) *versus* treatments

**Figure 8:**
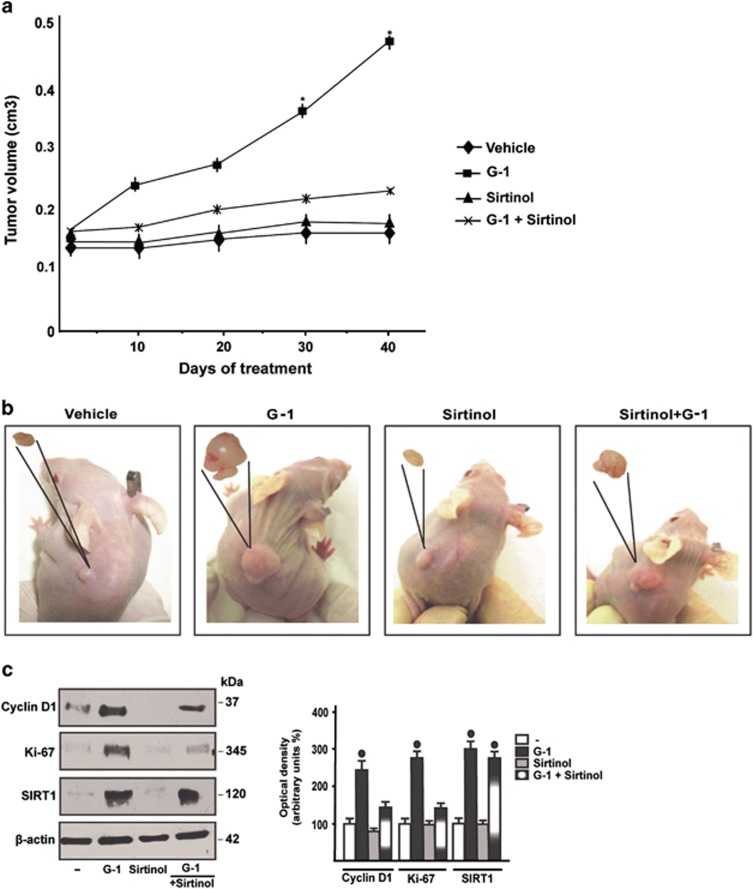
SIRT1 is involved in the growth of SkBr3 xenografts. (**a**) Tumor volume from SkBr3 xenografts implanted in female athymic nude mice treated for 40 days with vehicle, G-1 (0.50 mg/kg/die), Sirtinol (10 mg/kg/die) or a combination of these agents, as indicated. * indicates *P*<0.05 for animals treated with G-1 *versus* animals treated with vehicle. (**b**) Representative images of mice and relative explanted tumors at day 40, scale bar, 0.3 cm. (**c**) Cyclin D1, Ki-67 and SIRT1 protein levels in tumor homogenates from SkBr3 xenografts treated as reported above. Side panels show densitometric analysis of the blots normalized to *β*-actin. ^•^ indicates *P*<0.05 for G-1-treated animals *versus* vehicle-treated animals

In order to evaluate the potential of GPER along with SIRT1 to stimulate growth effects, we first assessed that in SkBr3 cells the induction of Cyclin D1 by E2 and G-1 is abolished silencing GPER expression, as well as in the presence of the DN/c-fos construct or Sirtinol (Figures 7a and e). In agreement with these results, the proliferation of SkBr3 cells upon exposure to E2 and G-1 was no longer evident of knocking down GPER expression (Figure 7f), in the presence of the DN/c-fos construct (Figure 7g) or Sirtinol (Figure 7h), as well as silencing SIRT1 expression (Figure 7i). Afterward, we evaluated the influence of SIRT1 on tumor growth *in vivo* in 45-day-old female nude mice bearing into the intrascapular region the SkBr3 cells. Tumor xenografts were treated with vehicle, G-1 at 0.5 mg/kg/day alone and in combination with Sirtinol at 10 mg/kg/day.^32, 33, 34^ These administrations were well tolerated as no change in body weight or in food and water consumption was observed together with no evidence of reduced motor function. No significant difference in the mean weights or histologic features of the major organs (liver, lung, spleen and kidney) was also detected after killing among vehicle and ligand-treated mice, thus indicating a lack of toxic effects. After 40 days of treatment, histologic examination of SkBr3 xenografts revealed that tumors explanted were primarily composed of human epithelial cells (Supplementary Figure 5). Moreover, we assessed that tumor growth induced by G-1 is prevented by Sirtinol (Figures 8a and b). Of note, increased Cyclin D1, Ki-67 and SIRT1 protein levels were found in tumor homogenates obtained from G-1 stimulated mice with respect to mice treated with vehicle, however, these stimulatory effects were prevented in the group of animals receiving G-1 in combination with Sirtinol (Figure 8c). Taken together, these results indicate that SIRT1 is also involved in tumor growth prompted by G-1 *in vivo*.

## Discussion

In the present study, we provide novel insights into the regulation and function of SIRT1 by estrogens in ER-negative breast cancer cells and CAFs. In particular, we demonstrate that E2 and the selective GPER agonist G-1 induce SIRT1 expression through the rapid activation of the EGFR/ERK1/2 signaling and the stimulation of c-fos expression which is recruited to the AP-1 site located within the SIRT1 promoter sequence. Noteworthy, GPER mediates the upregulation of SIRT1 by E2 and G-1, as ascertained by silencing experiments. Using the DNA damaging agent ETO, we also disclose that GPER along with SIRT1 are involved in the pro-survival effects elicited by these ligands, as demonstrated knocking down GPER expression and using the SIRT1 inhibitor Sirtinol. Biologically, we show that GPER and SIRT1 contribute to the growth effects triggered by E2 and G-1 *in vitro*, as well as in breast tumor xenografts. In accordance with these findings, Sirtinol abrogated the increase of both Cyclin D1 and the proliferative index Ki-67 upon G-1 treatment, as assessed in tumor homogenates. Collectively, our data reveal that SIRT1 may be engaged by GPER signaling toward tumor progression and pro-survival effects elicited by estrogens in cancer cells and main components of the tumor microenvironment like CAFs.

Sirtuins have drawn increasing attention due to their action in various patho-physiological processes as lifespan extension, aging, neurodegeneration, obesity, heart disease, inflammation and cancer.^16^ In mammals, the sirtuins family includes seven members (SIRT1-7) that show distinct structure, distribution and functions.^35^ SIRT1 is the mammalian homolog of the yeast silent information regulator 2 (sir2) and the most extensively studied sirtuins member.^16^ SIRT1 deacetylates several histone and non-histone proteins involved in the regulation of numerous cellular and metabolic processes including gene silencing, cell cycle progression, differentiation, apoptosis and aging.^17, 36, 37^ For instance, SIRT1 inactivates the tumor suppressor p53 deacetylating the Lys382 residue.^38, 39^ Inactive p53 then leads to a defective apoptotic response to DNA damage, suggesting that SIRT1 may contribute to cancer initiation and progression.^40^ Other SIRT1 downstream targets include NF-*κ*B, PPAR*-γ*, p63, p73, FOXO, Ku70 and the androgen receptor.^22, 39, 41, 42, 43^ To date, the function of SIRT1 remains controversial as previous data suggest that SIRT1 can act as a tumor promoter or a tumor suppressor likely depending on cell type, its distribution and biological targets.^19, 20, 21^ SIRT1-deficient mice developed tumors in many tissues^44^ and the overexpression of SIRT1 prevented intestinal tumorigenesis in transgenic mice,^45^ nevertheless SIRT1 activity was suggested to have a role in breast and prostate cancer cell growth.^46, 47^ In addition, SIRT1 was involved in oncogenic signaling in mammary epithelial cancer cells^48^ and SIRT1 knockout mice exhibited p53 hyperacetylation and increased apoptosis upon radiation exposure.^49^ SIRT1 was also shown to suppress senescence and apoptosis indicating that its inhibition may be beneficial in diverse types of cancer.^50, 51^ Consequently, a number of SIRT1 inhibitors have been identified in order to interfere with cell proliferation in various types of tumors.^19, 52, 53, 54, 55^

Estrogens exert diverse patho-physiological functions, including the development and maintenance of female reproductive system and the progression of breast cancer.^56^ The action of estrogens is mainly mediated by the classical ER, however, these steroids act also through GPER in both normal and malignant cell contexts, like breast cancer cells and CAFs that are main factors of the tumor microenvironment.^5, 8, 10, 11, 56, 57^ In particular, the stromal contribution to the development of a wide variety of tumors has been extensively assessed using both *in vitro* and *in vivo* model systems.^58, 59, 60^ For instance, it has been shown that malignant cells may recruit into the tumor mass diverse components of the microenvironment like CAFs, inflammatory and vascular cells that actively cooperate toward cancer progression.^58^ Increasing evidence has suggested that CAFs contribute to cancer aggressiveness through the production of secreted factors, which target numerous stromal components and cancer cell types.^59, 61^ In breast carcinoma ~80% of stromal fibroblasts exhibit the activated features of CAFs that stimulate the proliferation of cancer cells also at the metastatic sites.^62^ CAFs may also promote the local production of estrogens, which largely contribute to the development of breast carcinomas through an intricate cross-talk with many transduction pathways activated by growth factors.^63^ In addition, the ER antagonist tamoxifen was shown to upregulate the aromatase expression through GPER in both breast cancer cells and CAFs, suggesting that GPER may be involved in the tamoxifen resistance in breast cancer.^64^ In this context, our current results provide evidence regarding a novel mechanism by which estrogens through GPER engages SIRT1 toward the stimulation of breast cancer cells, CAFs and breast tumor xenografts. Previous studies have demonstrated that ER*α* is involved in cell survival and oncogenic transformation triggered by E2 via activation of anti-oxidative enzymes, MAPK, PI3-K and p53 inhibition.^18, 29^ In addition, it has been shown that ER*α* and SIRT1 actively cooperate in mediating the protection elicited by E2 against DNA damaging agents.^18^ Further extending these mechanisms of estrogen action, the current results indicate that E2 through GPER protect ER-negative breast cancer cells and CAFs from the DNA damage occurring upon ETO treatment. For instance, we have found that GPER and SIRT1 are involved in the prevention of cell cycle arrest and apoptosis prompted by ETO. Hence, GPER targets SIRT1 as ER*α* toward cell survival and tumor growth, suggesting that appropriate combination therapies could offer more effective interventions according to the ER expression pattern in breast cancer.

## Materials and Methods

### Materials

Tyrphostin AG1478 (AG) was purchased from Biomol Research Laboratories (Milan, Italy). PD98059 (PD) and Sirtinol were obtained from Calbiochem (Milan, Italy). 1-[4-(-6-Bromobenzol1,3diodo-5-yl)-3a,4,5,9btetrahydro-3H-cyclopenta[c−] quinolin8yl] ethanone (G-1) was purchased from Tocris Bioscience (Bristol, UK). E2, H89, LY294002 (LY) and ETO were purchased from Sigma-Aldrich Srl (Milan, Italy). All compounds were solubilized in dimethyl sulfoxide (DMSO), except E2 and PD which were dissolved in ethanol.

### Cell culture

SkBr3 and MCF-7 breast cancer cells and LNCaP prostate cancer cells were obtained by ATCC (Manassas, VA, USA) and used <6 months after resuscitation. SkBr3 and LNCaP were maintained in RPMI-1640 without phenol red, MCF-7 was maintained in DMEM medium, with a supplement of 10% fetal bovine serum (FBS; Sigma-Aldrich Srl) and 100 *μ*g/ml of penicillin/streptomycin (Life Technologies, Milan, Italy). CAFs obtained from breast cancer patients, were characterized and maintained as we previously described.^57, 65^ Signed informed consent from all the patients was obtained and all samples were collected, identified and used in accordance with approval by the Institutional Ethical Committee Board (Regional Hospital, Cosenza, Italy). All cell lines were grown in a 37 °C incubator with 5% CO2. Cells were switched to medium without serum 24 h before experiments.

### Gene silencing experiments and plasmids

Cells were plated onto 10-cm dishes and transfected by X-treme GENE 9 DNA transfection reagent (Roche Molecular Biochemicals, Milan, Italy) for 24 h before treatments with a control vector, a specific shRNA sequence for each target gene, the DN/c-fos construct which encodes for c-fos mutant that heterodimerizes with c-fos dimerization partners but not allowing DNA binding (kindly obtained from Dr C Vinson, NIH, Bethesda, MD, USA). The silencing of GPER expression was obtained by a construct (shGPER) previously described,^66^ whereas the silencing of SIRT1 expression was obtained by a construct (shSIRT1) kindly provided by Dr H Cha, (Sogang University, Seoul, Korea).

### Gene expression studies

Total RNA was extracted and cDNA was synthesized by reverse transcription as previously described.^13^ The expression of selected genes was quantified by real-time PCR using Step One sequence detection system (Applied Biosystems, Milan, Italy). Gene-specific primers were designed using Primer Express version 2.0 software (Applied Biosystems). For SIRT1, Cyclin D1 and the ribosomal protein 18 S, which was used as a control gene to obtain normalized values, the primers were: 5′-CTCTAGTGACTGGACTCCAAGG-3′ (SIRT1 forward), 5′-AAGATCTGGGAAGTCTACAGCA-3′ (SIRT1 reverse), 5′-GTCTGTGCATTTCTGGTTGCA-3′ (Cyclin D1 forward), 5′-GCTGGAAACATGCCGGTTA-3′ (Cyclin D1 reverse), 5′-GGCGTCCCCCAACTTCTTA-3′ (18 S forward) and 5′-GGGCATCACAGACCTGTTATT-3′ (18 S reverse). Assays were performed in triplicate and the results were normalized for 18 S expression and then calculated as fold induction of RNA expression.

### Western blot analysis

SkBr3 cells, CAFs and tumor homogenates obtained from nude mice were processed according to the previously described protocol.^67, 68, 69^ Protein lysates were electrophoresed through a reducing SDS/10% (w/v) polyacrylamide gel, electroblotted onto a nitrocellulose membrane probed with primary antibodies against SIRT1 (D739) and acetyl-p53 (Lys382) purchased from Cell Signaling Technology, Euroclone (Milan, Italy), c-fos (H-125), phosphorylated ERK1/2 (E-4), ERK2 (C-14), EGFR (1005), p-EGFR^Tyr1173^ (sc-12351), p21 (H164), GPER (N-15), Cyclin D1 (M-20), Ki-67 (H-300) and *β*-actin (C2) purchased from Santa Cruz Biotechnology (DBA, Milan, Italy). The levels of proteins and phosphoproteins were detected, after incubation with the horseradish peroxidase-linked secondary antibodies (Santa Cruz Biotechnology), by the ECL System (GE Healthcare, Milan, Italy).

### Chromatin immunoprecipitation (ChIP) assay

Cells grown in 10-cm plates were shifted for 24 h to medium lacking serum and then treated with vehicle, 100 nM E2 and 1 *μ*M G-1. Chip assay was performed as previously described.^70^ In brief, the immune-cleared chromatin was immunoprecipitated with anti-c-fos (H-125) or nonspecific IgG (Santa Cruz Biotecnology). A 4-*μ*l volume of each immunoprecipitated DNA sample was used as template to amplify, by real-time PCR, a region containing an AP-1 site located into the SIRT1 promoter region. The primers used to amplify this fragment were: 5′-GCTCACGCTAGAAAGGAAGG-3′ (forward) and 5′-GGAAGACCTTTGACGTGGAG-3′ (reverse). The data were normalized with respect to unprocessed lysates (input DNA). Inputs DNA quantification was performed by using 4 *μ*l of the template DNA. The relative antibody-bound fractions were normalized to a calibrator that was chosen to be the basal, untreated sample. Final results were expressed as percent differences with respect to the relative input.

### Gene reporter assays

The 2.2 kb SIRT1 promoter-luciferase construct containing full-length SIRT1 promoter sequence used in luciferase assays was a kind gift from Dr M Thangaraju, (Georgia Health Sciences University, Augusta, GA, USA). SkBr3 cells and CAFs (1 × 10^5^) were plated into 24-well dishes with 500 *μ*l/well culture medium containing 10% FBS and transfected for 24 h with control vector and DN/c-fos construct. A mixture containing 0.5 *μ*g of reporter plasmid and 10 ng of pRL-TK was then transfected by using X-treme GENE 9 DNA transfection reagent, as recommended by the manufacturer (Roche Diagnostics). After 8 h, cells were treated for 18 h with E2 and G-1 in serum-free medium. Luciferase activity was measured with Dual Luciferase Kit (Promega, Milan, Italy) and normalized to the internal transfection control provided by Renilla luciferase. The normalized relative light unit values obtained from cells treated with vehicle were set as onefold induction, upon which the activity induced by treatments was calculated.

### FACS analysis

Around 1 × 10^5^ cells per well were seeded into 12-well plates and maintained in medium for 24 h. For knockdown experiments, cells were transfected for 48 h with shRNA constructs directed against GPER and with an unrelated shRNA construct (3 *μ*g DNA/well transfected with X-treme GENE 9 DNA transfection reagent in medium without serum). Cells were then treated with 20 *μ*M ETO alone and in combination with 100 nM E2, as well as in presence of 25 *μ*M Sirtinol. After 8 h, cells were pelleted, washed once with phosphate buffered saline (PBS) and resuspended in 0.5 ml of a 50 *μ*g/ml propidium iodide in 1 × PBS (PI) solution containing 20 U/ml RNAse-A and 0.1% triton and incubated for 1 h (Sigma-Aldrich). Cells were analyzed for DNA content by FACS (BD, FACS JAZZ). Cell phases were estimated as a percentage of a total of 10 000 events.

### Tunel assay

SkBr3 cells and CAFs were seeded into coverslips and maintained in medium for 24 h. Next, cells were serum-deprived, transfected and treated as indicated. Therefore, cells were fixed in 4% buffered paraformaldehyde for 15 min. Slides were rinsed twice in PBS, pH 7.4. For the detection of DNA fragmentation at the cellular level, cells were stained using DeadEnd Fluorometric Tunel System (Promega) following the manufacturer's instructions. Nuclei of cells were stained with 4′,6-Diamidino-2-phenylindole dihydrochloride (DAPI; 1 : 1000; Sigma-Aldrich). The Leica AF6000 Advanced Fluorescence Imaging System supported by quantification and image processing software Leica Application Suite Advanced Fluorescence (Leica Microsystems CMS, GmbH Mannheim, Germany) was used for the microscopy evaluation.

### Proliferation assay

For quantitative proliferation assay, SkBr3 cells (1 × 10^5^) were seeded in 24-well plates in regular growth medium. Cells were washed once they had attached and then incubated in medium containing 2.5% charcoal-stripped FBS with the indicated treatments; medium was renewed every 2 days (with treatments) and cells were counted using the Countess Automated Cell Counter, as recommended by the manufacturer's protocol (Life Technologies).

### *In vivo* studies

Female 45-day-old athymic nude mice (nu/nu Swiss; Harlan Laboratories, Milan, Italy) were maintained in a sterile environment. At day 0, exponentially growing SkBr3 cells (8.0 × 10^6^ per mouse) were inoculated into the intrascapular region in 0.1 ml of Matrigel (Cultrex, Trevigen, Gaithersburg, MD, USA). When the tumors reached average ~0.15 cm^3^ (i.e., in about 1 week after implantation), mice were randomized and divided into four groups, according to treatments administered by intramuscular injection for 40 days. The first group of mice (*n*=7) was treated daily with vehicle (0.9% NaCl with 0.1% albumin and 0.1% Tween-20), (Sigma-Aldrich), the second group of mice (*n*=7) was treated daily with G-1 (0.5 mg/kg/die), the third group of mice (*n*=7) was treated daily Sirtinol (10 mg/kg/die) and the fourth group of mice (*n*=7) was treated daily with G-1 in combination with Sirtinol (at the concentrations described above). G-1 and Sirtinol were dissolved in DMSO at 1 mg/ml. SkBr3 xenograft tumor growth was monitored twice a week by caliper measurements, along two orthogonal axes: length (L) and width (W). Tumor volumes (in cubic centimeters) were estimated by the following formula: TV=L × (W2)/2. At 40 days of treatment, the animals were killed following the standard protocols and tumors were dissected from the neighboring connective tissue. Specimens of tumors were frozen in nitrogen and stored at –80 °C; the remaining tumor tissues of each sample were fixed in 4% paraformaldehyde and embedded in paraffin for the histologic analyses. Animal care, death and experiments were done in accordance with the US National Institutes of Health Guide for the Care and Use of Laboratory Animals (NIH Publication No 85–23, revised 1996) and in accordance with the Italian law (DL 116, 27 January 1992).

### Histologic analysis

Morphologic analyses were carried out on formalin-fixed, paraffin-embedded sections of tumor xenografts were cut at 5 *μ*m and allowed to air dry. Deparaffinized, rehydrated sections were stained with hematoxylin and eosin (Bio-Optica, Milan, Italy) or immunolabeled with human cytocheratin 18 (Santa Cruz Biotechnology) to verify that the tumors explanted will be primarily composed of human epithelial cells. Sections were then dehydrated, cleared with xylene, and mounted with resinous mounting medium.

### Statistical analysis

Statistical analysis was performed using ANOVA followed by Newman-Keuls' testing to determine differences in means. Statistical comparisons for *in vivo* studies were made using the Wilcoxon–Mann–Whitney test. *P*<0.05 was considered statistically significant.

## Abbreviations

GPER: G-protein estrogen receptor
CAFs: cancer-activated fibroblasts
SIRT1: silent information regulator 1
EGFR: epidermal growth factor receptor
ERK: extracellular signal-regulated kinase
AP-1: activator protein 1
HDACs: histone deacetylases
