# Supplementary material for: SIRT1 is involved in oncogenic signaling mediated by GPER in breast cancer
Source: Cell Death Dis. 2015 Jul 30;6(7):e1834–. doi: 10.1038/cddis.2015.201 (PMC4650744; doi:10.1038/cddis.2015.201)
Supplement: Supplementary Information [file cddis2015201x1.doc]

**Supplementary information**

**Supplementary Figure 1.** ERα and ERβ mRNA (a) and protein (b) expression in SkBr3, CAFs, MCF-7 and LNCaP cells, as evaluated by real-time PCR and immunoblotting. In RNA experiments, PCR amplification in absence of cDNA was used as a control (-) and each data point represents the mean ±SD of three independent experiments performed in triplicate. In immunoblot assays β-actin was used as loading control.

**Supplementary Figure 2.** Efficacy of GPER silencing obtained transfecting SkBr3 cells and CAFs with a shGPER construct. Side panels show densitometric analysis of the blots normalized to β-actin. (○) indicates p<0.05 for cells receiving vehicle (-) versus treatments.

**Supplementary Figure 3. A**poptotic changes detected using Tunel (green) and DAPI (blu) staining upon treatment for 24h with 20μM etoposide (ETO) alone and in combination with 100nM E2 and 25μM Sirtinol in CAFs transfected with shRNA (a) or shGPER (b). Each experiment shown is representative of 20 random fields. Data are representative of three independent experiments**.**

**Supplementary Figure 4.** Apoptosis changes were detected using Tunel (green) and DAPI (blu) staining in SkBr3 cells transfected with shRNA (a) or shSIRT1 (b), upon a 24h treatment with 20μM etoposide (ETO) alone and in combination with 100nM E2. Each experiment shown is representative of 20 random fields. Data are representative of three independent experiments**.**

**Supplementary Figure 5.** Staining of SkBr3 tumor xenografts. Representative images from tumors that at the end of the treatments (40 days) were formalin fixed, paraffin embedded, sectioned and stained with hematoxylin and eosin Y (H&E) (a) or incubated with a mouse monoclonal antibody directed against human cytokeratin 18 which appears as brown cytoplasmic staining (b).
