# Supplementary figures and images for: SIRT1 is involved in oncogenic signaling mediated by GPER in breast cancer
Source: Cell Death Dis. 2015 Jul 30;6(7):e1834–. doi: 10.1038/cddis.2015.201 (PMC4650744; doi:10.1038/cddis.2015.201)

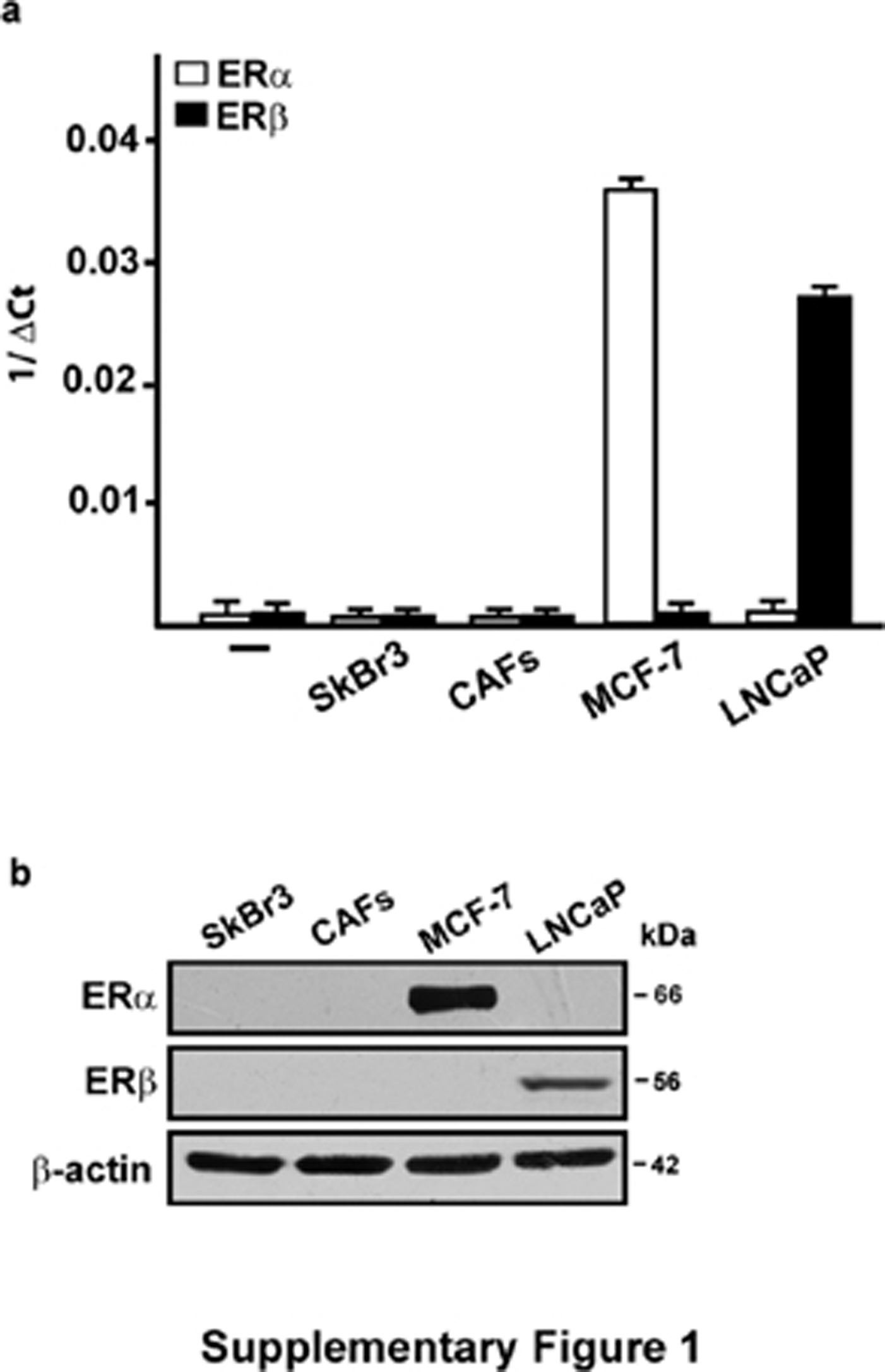

Supplement: Supplementary Figure 1 [file cddis2015201x2.tif]

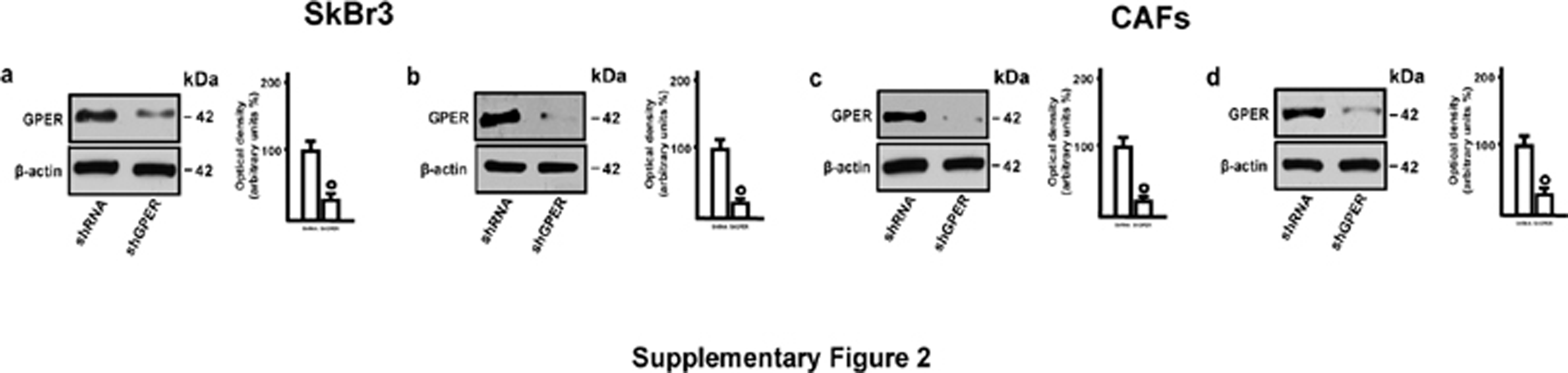

Supplement: Supplementary Figure 2 [file cddis2015201x3.tif]

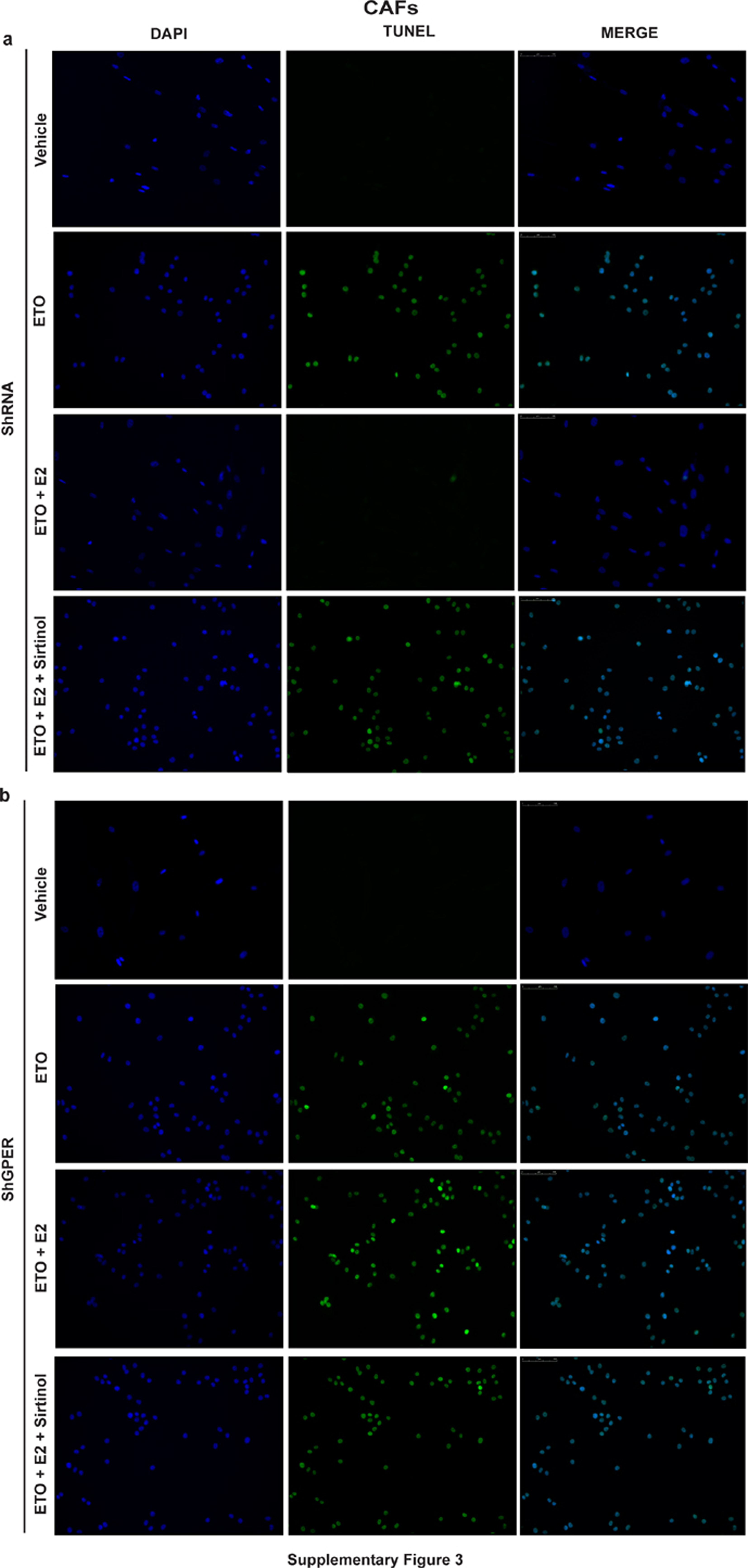

Supplement: Supplementary Figure 3 [file cddis2015201x4.tif]

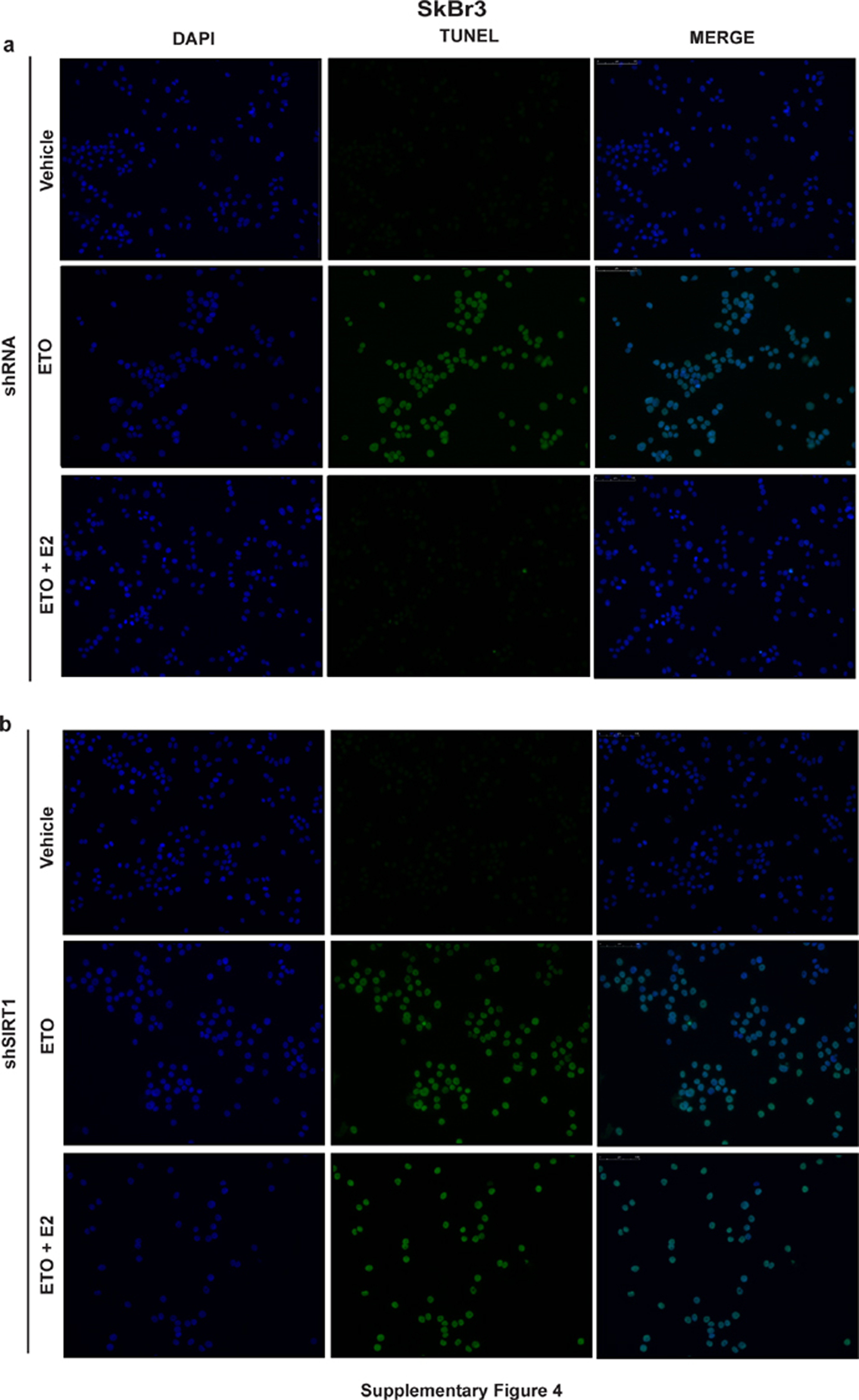

Supplement: Supplementary Figure 4 [file cddis2015201x5.tif]

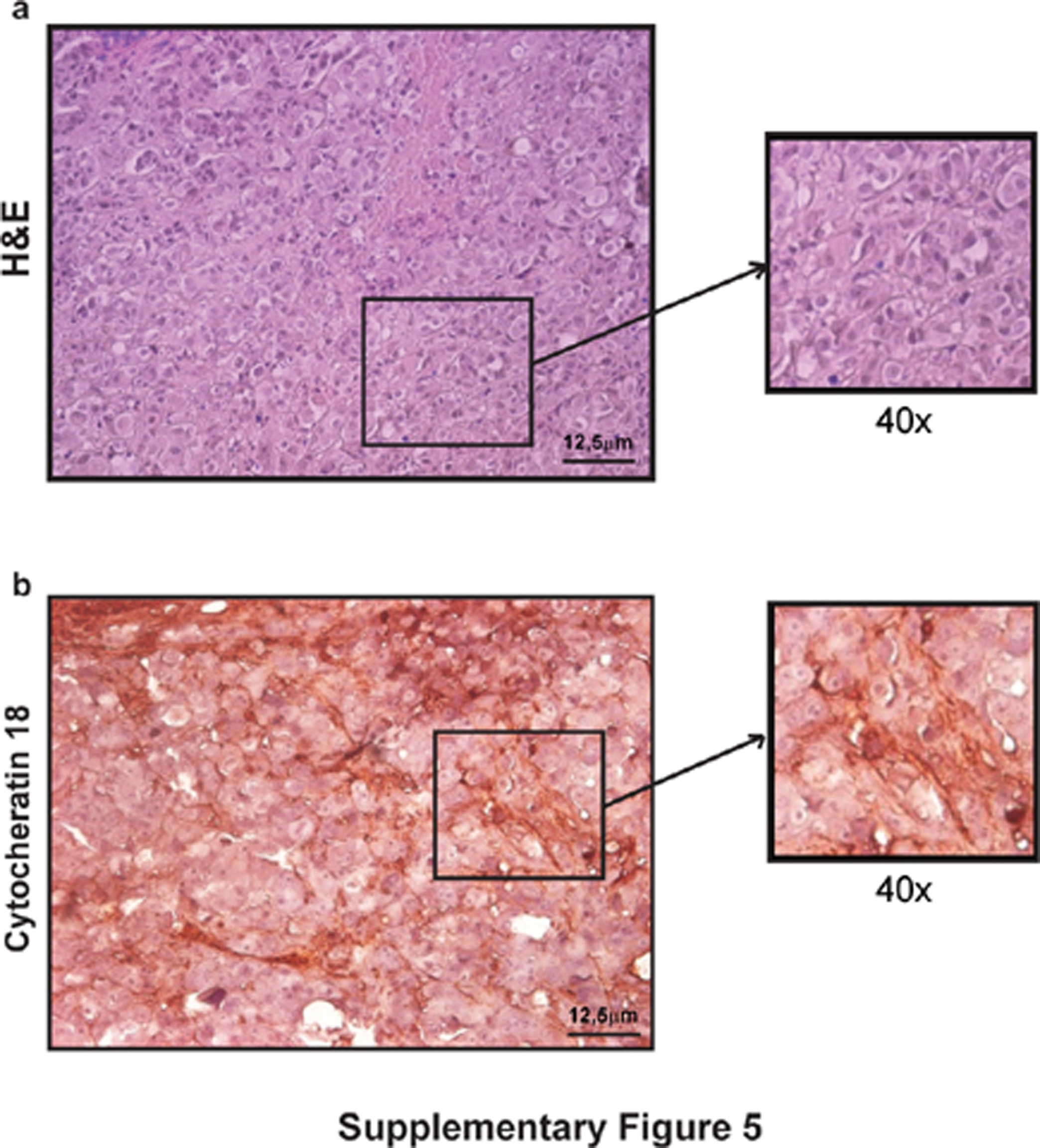

Supplement: Supplementary Figure 5 [file cddis2015201x6.tif]
